# Harmonic motion imaging of human breast masses: an in vivo clinical feasibility

**DOI:** 10.1038/s41598-020-71960-5

**Published:** 2020-09-17

**Authors:** Niloufar Saharkhiz, Richard Ha, Bret Taback, Xiaoyue Judy Li, Rachel Weber, Alireza Nabavizadeh, Stephen A. Lee, Hanina Hibshoosh, Vittorio Gatti, Hermes A. S. Kamimura, Elisa E. Konofagou

**Affiliations:** 1 Department of Biomedical Engineering, Columbia University, New York, NY USA; 2 Department of Radiology, New-York-Presbyterian/Columbia University Medical Center, New York, NY USA; 3 Department of Surgery, New-York-Presbyterian/Columbia University Medical Center, New York, NY USA; 4 Department of Pathology and Cell Biology, New-York-Presbyterian/Columbia University Medical Center, New York, NY USA

**Keywords:** Ultrasound, Breast cancer, Biomedical engineering, Breast cancer, Translational research

## Abstract

Non-invasive diagnosis of breast cancer is still challenging due to the low specificity of the imaging modalities that calls for unnecessary biopsies. The diagnostic accuracy can be improved by assessing the breast tissue mechanical properties associated with pathological changes. Harmonic motion imaging (HMI) is an elasticity imaging technique that uses acoustic radiation force to evaluate the localized mechanical properties of the underlying tissue. Herein, we studied the in vivo feasibility of a clinical HMI system to differentiate breast tumors based on their relative HMI displacements, in human subjects. We performed HMI scans in 10 female subjects with breast masses: five benign and five malignant masses. Results revealed that both benign and malignant masses were stiffer than the surrounding tissues. However, malignant tumors underwent lower mean HMI displacement (1.1 ± 0.5 µm) compared to benign tumors (3.6 ± 1.5 µm) and the adjacent non-cancerous tissue (6.4 ± 2.5 µm), which allowed to differentiate between tumor types. Additionally, the excised breast specimens of the same patients (n = 5) were imaged post-surgically, where there was an excellent agreement between the in vivo and ex vivo findings, confirmed with histology. Higher displacement contrast between cancerous and non-cancerous tissue was found ex vivo, potentially due to the lower nonlinearity in the elastic properties of ex vivo tissue. This preliminary study lays the foundation for the potential complementary application of HMI in clinical practice in conjunction with the B-mode to classify suspicious breast masses.

## Introduction

Breast cancer is the most frequently diagnosed malignancy, excluding skin, in women with a lifetime risk of 12.4% in the United States. Due to the advances in diagnosis and treatment technologies, the mortality rate for breast cancer has dropped by 40% from 1989 to 2016. However, it is still estimated to be the second leading cause of cancer death^1^. Early detection and diagnosis of breast cancer are of crucial significance in improving survival and prognosis^2^.

The current gold standard imaging modalities are mammography, magnetic resonance imaging (MRI) or ultrasonography (US). Mammography has been shown to reduce breast cancer mortality^3,4^. However, the sensitivity of the technique declines significantly in women with dense breasts^5^. High breast density itself increases the risk of developing breast cancer^6^. This issue affects more than 50% of American women^7^. For high-risk patients, contrast-enhanced MRI is recommended as an adjunct to mammography because MRI has the highest sensitivity compared to other breast screening modalities^8^. However, the lower specificity of MRI results in more recalls and biopsies^8,9^. Ultrasonography is another supplemental screening method to mammography for patients who cannot undergo MRI^10^. Some ultrasound features that characterize tumors as malignant include shape irregularity, micro-lobulated or speculated margins, width-to-anteroposterior (AP) ratio, marked hypoechogenicity, and shadowing^11,12^. Hand-held ultrasound used by an experienced technologist or radiologist is effective in detecting mammographically invisible cancers in dense breasts^13–17^. A large multi-institutional trial reported that combined ultrasound with mammography increased the cancer detection yield by 4.2 cancers per 1,000 women^18^. Nevertheless, US can result in a higher false-positive rate compared to mammography and MRI^9,19^. Recently, automated breast ultrasound (ABUS) has been demonstrated to reduce the amount of time needed for image acquisition and interpretation, as well as to be effective in eliminating the operator dependence of image quality and reproducibility. However, inadequate axillary breast tissue imaging and artifacts caused by the nipple are limitations associated with the ABUS systems^20,21^. Thus, there is still a need for additional breast screening to reduce the false positive rate and subsequent unnecessary biopsies as only 20–40% of breast biopsies are cancerous^8^. An ideal breast screening tool must be non-invasive with high sensitivity and specificity and not be affected by breast density. In addition, the cost is an important factor in evaluating its likely extent of routine adoption.

Breast tissue hardness can be indicative of malignancy since the stiffness or Young’s modulus of cancerous tumors is higher than that of normal tissue^22–24^. However, soft and hard tumors do not necessarily appear with different echogenicity on conventional B-mode US. Ultrasound elasticity imaging techniques are complementary modalities to B-mode US to improve tissue characterization by providing information on the viscoelastic properties of the underlying tissue, based on tissue perturbation as a response to a mechanical stimulus. The perturbation source can be applied externally, as in transient elastography^25^ or manually by compressing a hand-held ultrasound transducer, as in strain elastography (SE)^26^. However, external excitation methods are impacted by multiple factors such as the external boundaries, the coupling of the excitation with the targeted region, and the amount of stress applied by the operator^27,28^. In addition, the image depth is limited by the attenuation and interaction of the signal with different tissue layers^28^. The tissue perturbation can also be applied internally using an acoustic radiation force (ARF). Several modalities have been introduced based on ARF perturbation using an imaging ultrasound transducer, including shear wave elasticity (SWE) imaging^29^, acoustic radiation force impulse (ARFI) imaging^30–32^, supersonic shear imaging (SSI)^33^ and comb-push ultrasound shear elastography (CUSE)^34^. The ARF generated by the imaging transducers may limit the depth of penetration and specificity due to the power limits of the imaging transducers and attenuation of the vibration energy through different layers of tissue. In fact, it has been shown that lesion size, breast thickness, and lesion depth are among the factors that may lead to false findings in ARFI shear wave imaging^35^. A comparison study between histopathology and ARFI elasticity imaging results has shown a 59.1% false positivity^36^. Vibro-acoustography is a novel ARF-based elasticity imaging technique in which two confocal ultrasound beams generate a low-frequency oscillating force, and the resulting tissue displacements are detected using a hydrophone^37,38,39,40^. Tissue stiffness estimation, however, is challenging since the hydrophone’s signal is affected by various factors, including the mechanical and acoustical properties of the tissue^41,42^.

Harmonic motion imaging (HMI) is an ultrasound-based elasticity imaging technique that assesses tissue viscoelastic properties by inducing periodic oscillations^41,43^. HMI uses a focused ultrasound (FUS) transducer to generate an amplitude-modulated (AM) ARF at its focal zone. The resulting tissue oscillations are estimated using a confocally aligned imaging transducer, and the recorded radiofrequency (RF) data is used to estimate the displacements. HMI overcomes some of the limitations associated with the existing elasticity imaging modalities. The oscillation energy generated by the FUS is high enough to penetrate deep inside the body and vibrate lesions with a wide range of stiffness, from normal soft tissues to "rock-like" tumors^44,45^. By using a separate imaging transducer to estimate the displacements, HMI is not affected by the acoustic properties and acoustic noise during signal acquisition^41^. The induced displacements are proportional to the AM frequency (Hz-range), which makes HMI more robust to low-frequency respiratory, cardiac, or body movement artifacts than impulsive radiation force imaging techniques^46^. A high special resolution is achieved as the primary beams are in the MHz-range, which generates a focal spot size of a few millimeters. The small region of oscillatory motion and low amplitudes of the deformations (of the order of micrometers) render HMI as a highly focal mechanical source technique. Thus, the amplitude of the HMI displacements is more directly related to the localized mechanical properties of the underlying tissue if the tumors are larger than twice the axial focal dimension^41,47^. In previous studies, HMI has shown to have promising potential in detection, characterization, and treatment assessment of solid tumors. Payen et al. demonstrated the feasibility of HMI for the assessment of disease staging and treatment response in pancreatic ductal adenocarcinoma in the mouse models as well as in resected human pancreatic cancer specimens^44^. Han et al. showed differentiation between normal and pathological tissues and monitoring of high intensity focused ultrasound (HIFU) ablation in human lumpectomy^48^. More recently, HMI has been shown capable of detecting breast tumors, and their response to neoadjuvant therapy in post-surgical mastectomy samples^49^.

This study aimed to develop a clinical setup to assess the feasibility of HMI in distinguishing human benign from malignant breast tumors. The pre-clinical setup that had been used in previous studies was improved and modified to facilitate the clinical application of HMI. Female patients with breast lesions were imaged with the clinical HMI setup before US-guided biopsy or breast surgery. RF data were acquired during a 1-D point-by-point mechanical raster scan using a robotic arm. Post-surgical breast tissues harvested from patients who underwent breast mastectomy or lumpectomy were scanned with a similar setup for comparison. The differentiation of breast lesions from normal breast tissue was evaluated offline using the peak-to-peak amplitude of HMI displacements.

## Results

In total, HMI images were acquired from 10 female patients with breast tumors. The pre-surgery subjects were scanned twice, before and after general anesthesia, intraoperatively to evaluate the effect of breathing, as well as reproducibility of the HMI measurements (Fig. S1). The HMI displacements reported in this study are the mean ± standard deviation and are averaged within a 5-mm circular ROI selected manually within the breast tumor and surrounding tissues at the same depth where the focus of the FUS transducer was placed. Clinical US images are included to support our results further and to delineate the anatomical border of tumors with higher resolution. Peak-to-peak-amplitude of HMI displacements is coded with red as high (soft) and blue as low (stiff) displacements.

**Figure 1:**
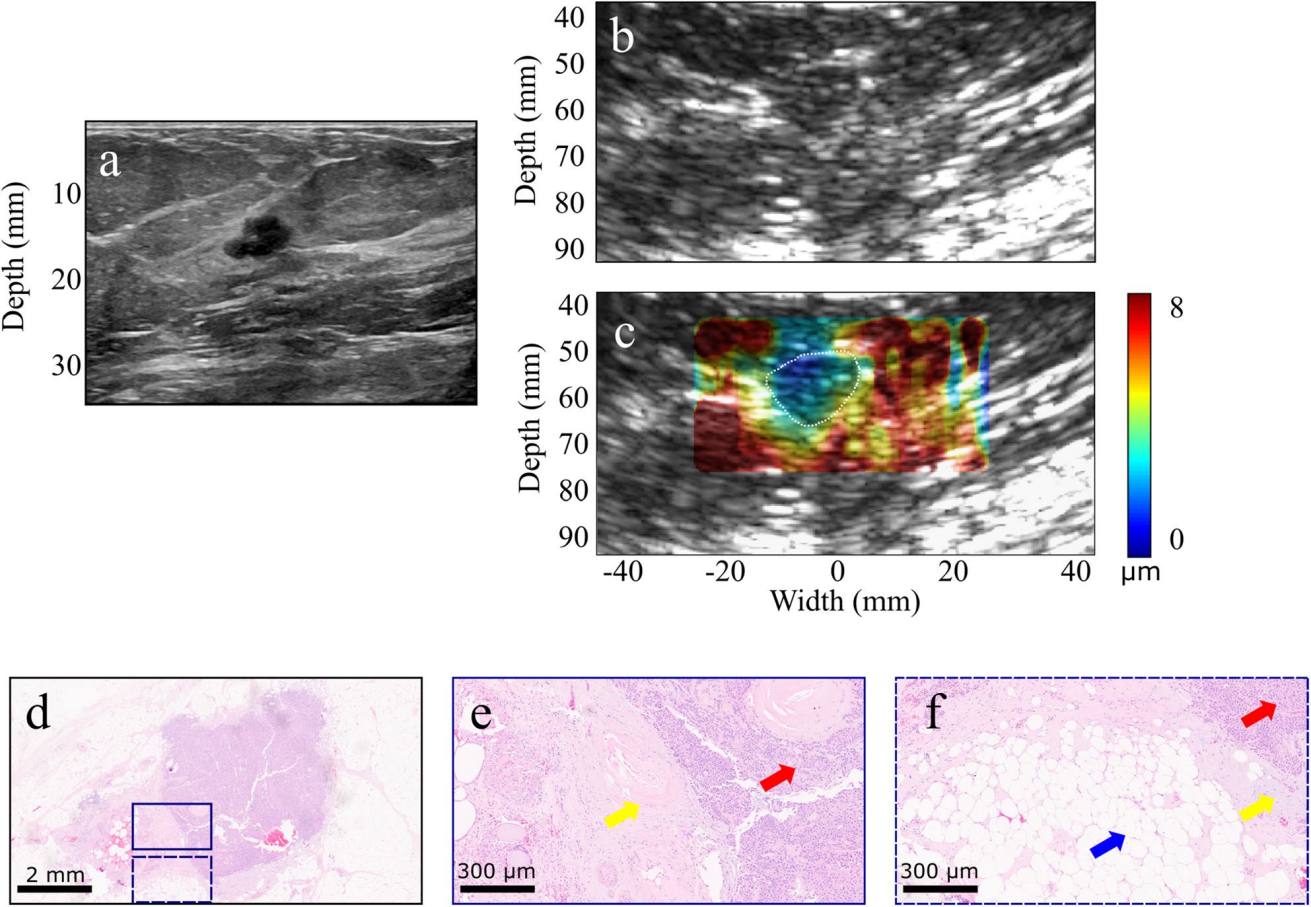
(**a**) Clinical B-mode image of a patient with a 0.9-cm invasive ductal carcinoma (IDC). (**b**) B-mode image of the mass acquired with the HMI imaging transducer (2.5 MHz). (**c**) HMI displacement map overlaid on the B-mode image (tumor contour is shown with white-dashed lines). (**d**–**f**) H&E staining of the mass. The red arrows show invasive carcinoma, the yellow arrows show fibrous normal breast tissue, and the blue arrow shows mature adipose tissue.

A clinical B-mode image of a recurrent 0.9-cm invasive ductal carcinoma (IDC) is shown in Fig. 1a. The 72-year-old patient was diagnosed with IDC in her 40s and underwent chemotherapy, radiation, and lumpectomy on the same breast. The B-mode image acquired with the HMI imaging probe is shown in Fig. 1b. The mean HMI displacements in the non-cancerous tissues and malignant tumor were estimated at 6.4 ± 0.6 µm and 1.9 ± 0.3 µm, respectively (Fig. 1c). The representative hematoxylin and eosin (H&E) staining of the mass and surrounding non-cancerous tissues are shown in Fig. 1d–f.

**Figure 2:**
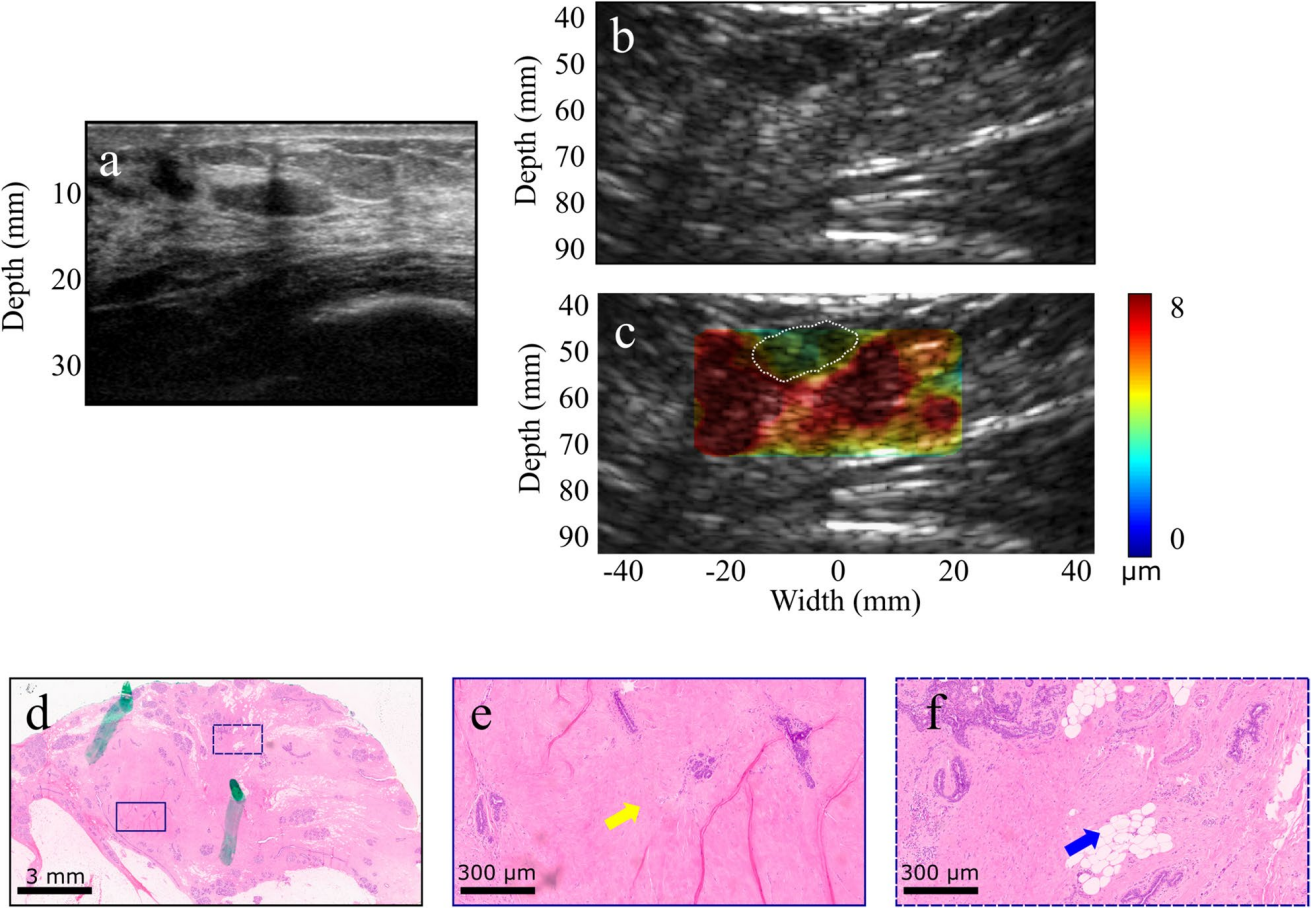
(**a**) Clinical B-mode image of a hypoechoic 1.4 cm solid breast mass. (**b**) B-mode image of the mass acquired with the HMI imaging transducer (2.5 MHz). (**c**) HMI displacement map overlaid on the B-mode image (tumor contour is shown with white-dashed lines). (**d**–**f**) H&E staining of the mass diagnosed as fibrotic stroma with adenosis. The yellow arrows show adenosis, and the blue arrow shows fibrous normal breast tissue and mature adipose tissue.

A 52-year-old female patient with a 1.4-cm hypoechoic solid mass in her left breast and a history of right breast lobular carcinoma was imaged before the biopsy. The clinical US image is shown in Fig. 2a and representative image from 2.5 MHz B-mode is shown in Fig. 2b. The mean HMI displacement amplitudes were equal to 4.5 ± 0.9 µm and 10.1 ± 1.3 µm in the breast tumor and surrounding tissues, respectively (Fig. 2c). Histopathological evaluation revealed that the mass was composed of fibrotic stroma with adenosis (Fig. 2d–f).

**Figure 3:**
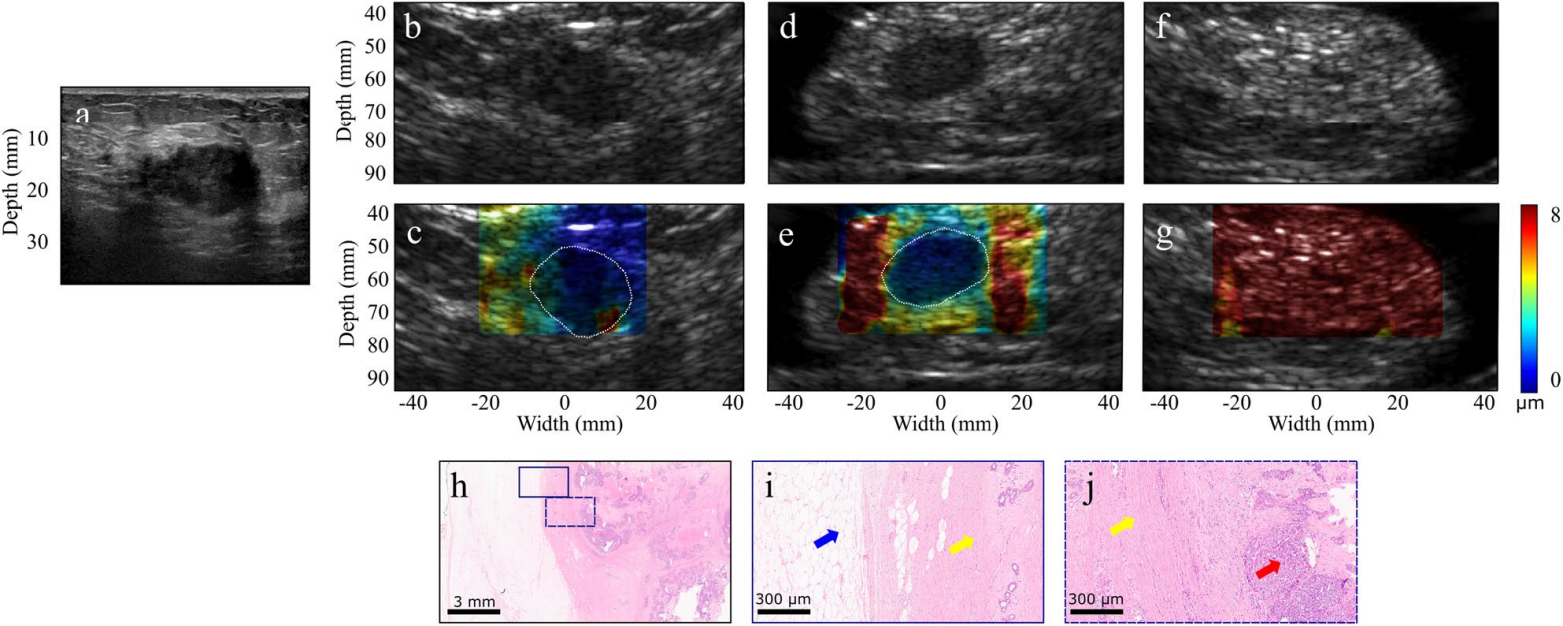
(**a**) US image of a 2.2-cm microlobulated solid hypoechoic invasive ductal carcinoma. (**b**–**c**) HMI B-mode image and displacement map of the in vivo tumor acquired before surgery (tumor contour is shown with white-dashed lines). (**d**–**e**) HMI B-mode image and displacement map of the tumor lumpectomy specimen scanned immediately after surgery. The contrast difference between the ex vivo displacement map and the corresponding in vivo map shown in (**c**) might be due to the change in the boundary conditions and lack of physical constraints in the ex vivo specimen. (**f**–**g**) HMI B-mode image and displacement map of the specimen at an imaging plane distant from the tumor. (**h**–**j**) H&E-stained sections. The red arrows show invasive carcinoma, the yellow arrows show fibrous normal breast tissue, and the blue arrow shows mature adipose tissue.

A patient in her 80s diagnosed with IDC associated with focal ductal carcinoma in situ (DCIS) in her right breast was scanned before lumpectomy. Of note, no evidence of malignancy was noticed on her mammogram dated 6 months before the cancer diagnosis. Figure 3a shows the clinical image where a 2.2-cm micro-lobulated solid hypoechoic mass is seen. The 2.5-MHz B-mode image of the in vivo tumor is shown in Fig. 3b. The tumor exhibited a mean HMI displacement of 0.8 ± 0.1 µm within the tumor, whereas the mean displacement in the non-cancerous surrounding tissues was 3.4 ± 0.6 µm (Fig. 3c). The mastectomy specimen was imaged immediately after resection (the B-mode image of the ex vivo tumor is shown in Fig. 3d) following the steps described in the Methods section. Higher contrast was found between the HMI displacements within the tumor and surrounding tissues, 1.3 ± 0.1 µm, and 4.8 ± 0.8 µm, respectively (Fig. 3e). A separate off-plane scan was performed on the ex vivo non-cancerous (5 cm from the tumor) (Fig. 3f), exhibiting significantly higher displacements than the tumor (Fig. 3g). The H&E staining of the mass and surrounding non-cancerous tissues are shown in Fig. 3h–j.

**Figure 4:**
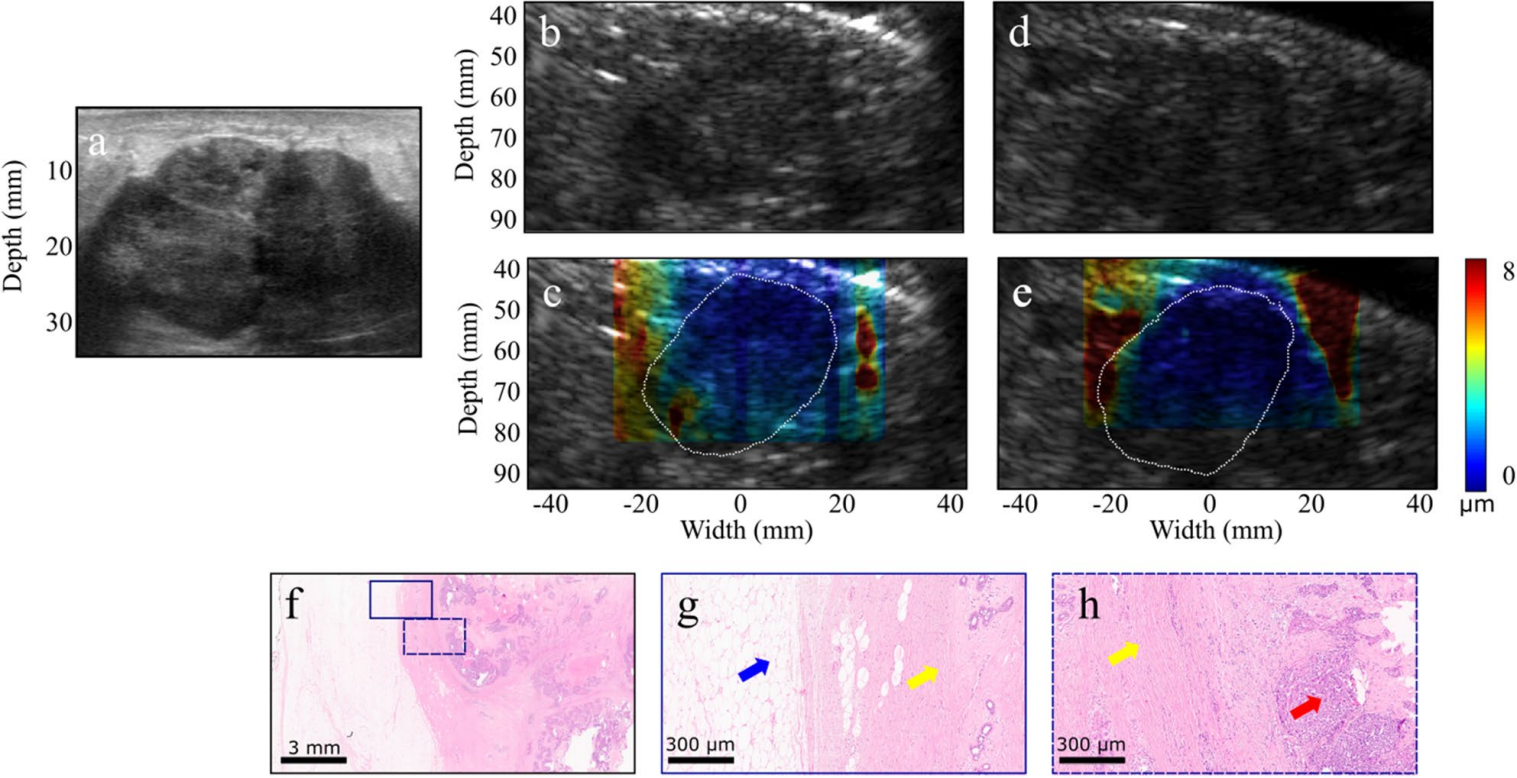
(**a**) A 4-cm invasive ductal carcinoma US image acquired using a clinical scanner (**b**–**c**) B-mode image and overlaid HMI displacement map of the in vivo tumor respectively before surgical resection (tumor contour is shown with white-dashed lines). (**d**–**e**) B-mode image and overlaid HMI displacement map of the ex vivo tumor respectively after surgical resection. (**f**–**h**) H&E-stained sections. The red arrows show invasive carcinoma, the yellow arrows show fibrous normal breast tissue, and the blue arrow shows mature adipose tissue.

In another case, the clinical and 2.5-MHz B-mode images of a 53-year old patient with 4-cm IDC in her left breast are shown in Fig. 4a–b. HMI was performed post-neoadjuvant chemotherapy and prior to surgery. HMI displacements were estimated as 0.9 ± 0.1 µm and 4.8 ± 0.5 µm in the carcinoma and peripheral non-cancerous tissues, respectively (Fig. 4c). The resected mastectomy specimen was scanned with the same acquisition parameters. The B-mode image and the 2-D reconstructed HMI displacement map are shown in Fig. 4d–e. Histopathological assessment of the specimen proved the mass to be metastatic breast carcinoma to axillary lymph nodes associated with extensive central necrosis (Fig. 4f–h).

**Figure 5:**
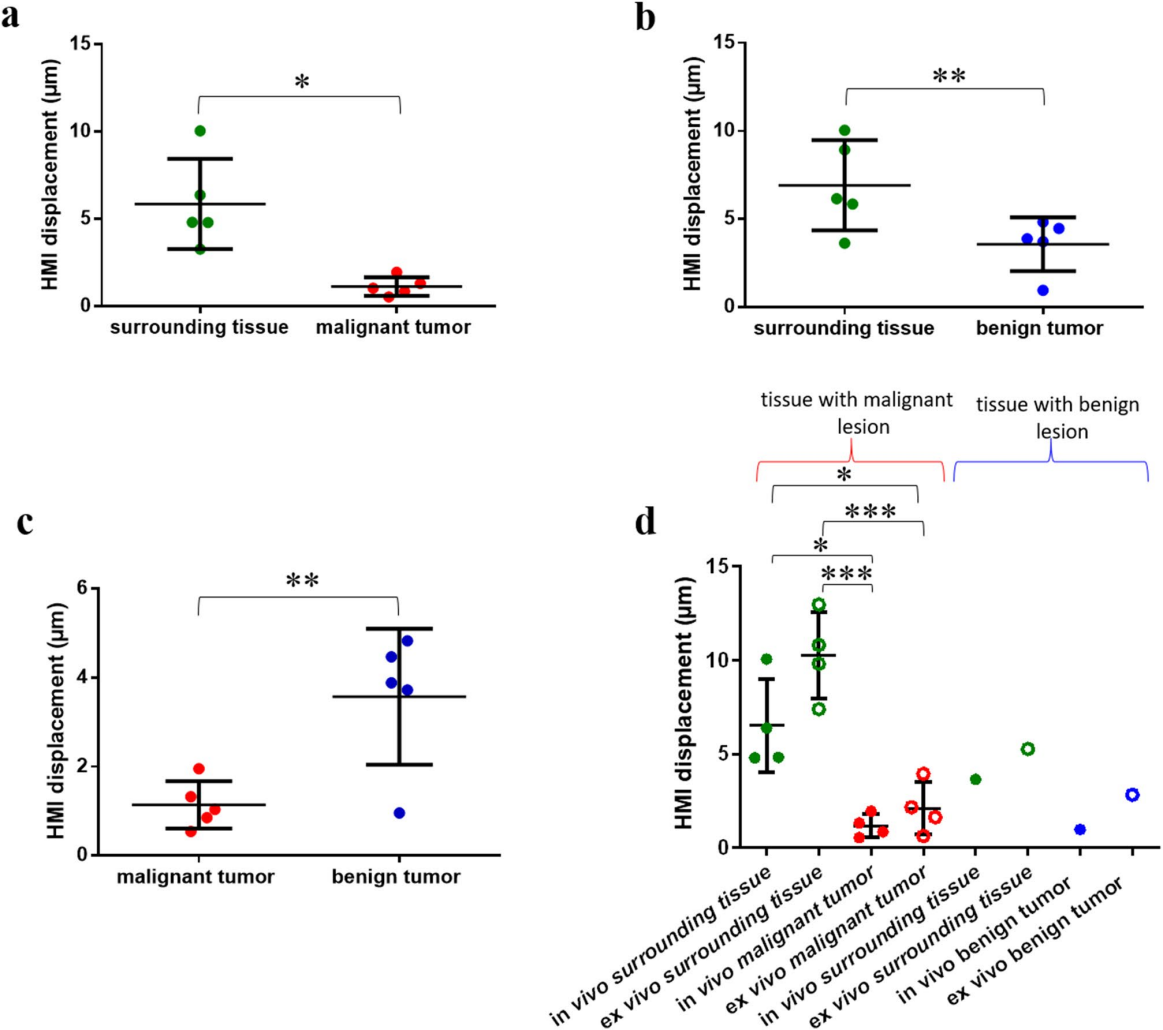
Harmonic motion imaging (HMI) displacement estimated in (**a**) non-cancerous tissue and tumor in five patients with malignant lesions, (**b**) surrounding tissue and tumor in five patients with benign lesions, (**c**) malignant (n = 5) and benign (n = 5) tumors in vivo*,* (**d**) non-cancerous tissue and tumor in patients with malignant (n = 4) and benign (n = 1) masses, in vivo and ex vivo. *p < 0.05, **p < 0.01 and ***p < 0.001.

**Figure 6:**
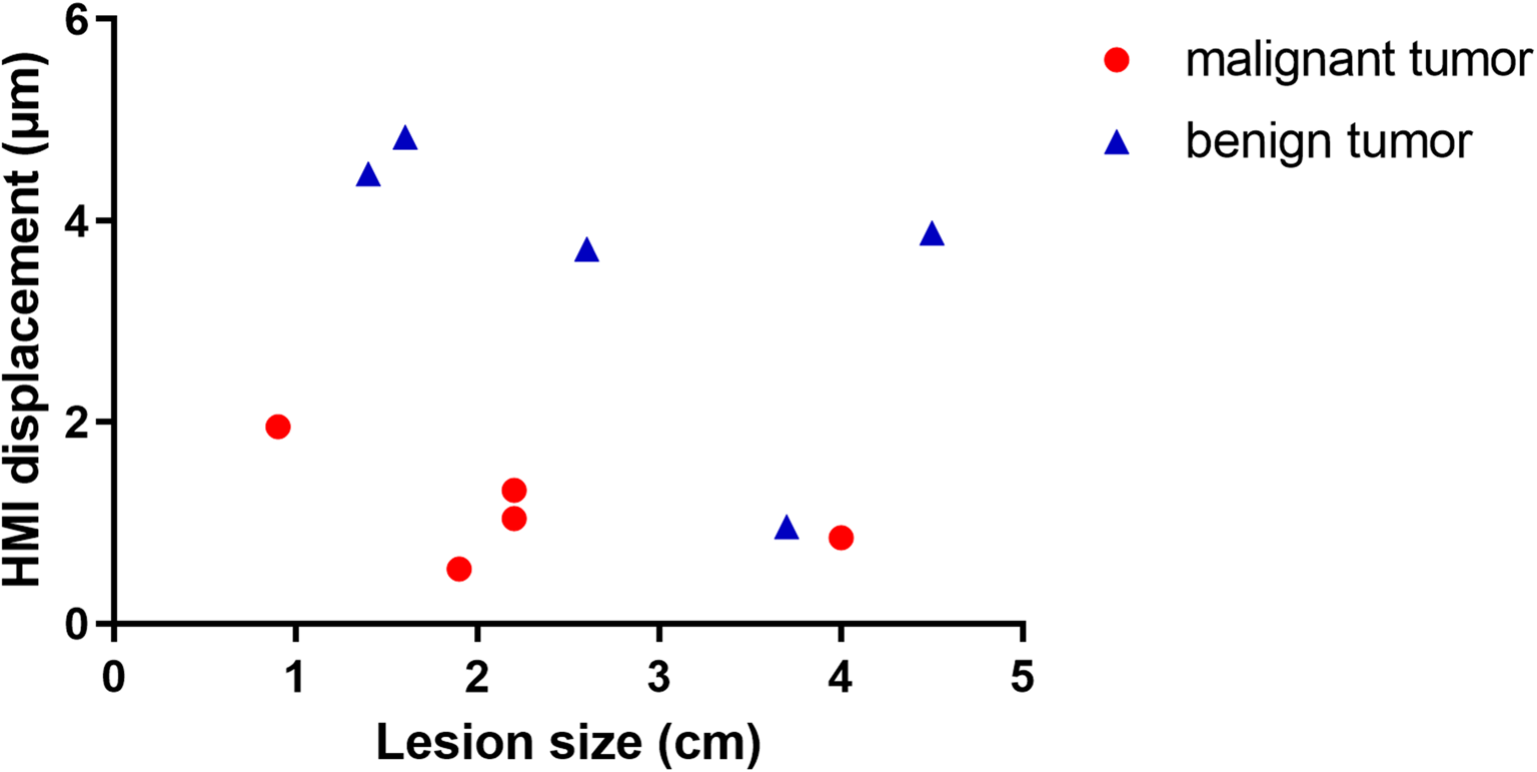
Estimated HMI displacement in different size tumors. No correlation was found between the displacement and tumor size (Pearson r = −0.1588, P = 0.66).

According to the HMI images acquired from the patients with malignant tumors (n = 5), the mean HMI displacement of the peripheral non-cancerous tissues (5.9 ± 2.6 µm) was significantly higher (P < 0.05) than that in the malignant tumors (1.1 ± 0.5 µm) (Fig. 5a). Similarly in the patients with benign tumors (n = 5), the mean HMI displacement in the surrounding tissues was found to be significantly higher (P < 0.01) than that in the benign tumors (6.9 ± 2.6 µm vs. 3.6 ± 1.5 µm) (Fig. 5b). A comparison between the mean HMI displacement within the malignant tumors (n = 5) versus benign tumors (n = 5) also showed a significant difference (P < 0.01) (Fig. 5c). No significantly different mean HMI displacement was found in non-cancerous tissues in vivo compared to ex vivo (5.9 ± 2.6 µm and 9.6 ± 3 µm, respectively) or in the malignant tumors before and after resection (1.1 ± 0.5 µm and 2.2 ± 1.2 µm, respectively)(Fig. 5d). The relationship between the HMI displacement and the lesion size was evaluated for all the in vivo benign and malignant masses (Fig. 6), and no correlation between the two variables was found (Pearson r =—0.1588, P = 0.66). The contrast-to-noise ratio (CNR), the ratio of the mean HMI displacement in the pathological tissue to that of the normal tissue $({\mu }_{T}/{\mu }_{S}),$ and correlation coefficients for the in vivo measurements are shown in Table 1. The CNR metric was computed as $CNR=\left|{\mu }_{S}-{\mu }_{T}\right|/\sqrt{{\sigma }_{S}^{2}+{\sigma }_{T}^{2}}$, where ${\upmu }_{\mathrm{S}}$, ${\upmu }_{\mathrm{T},}$ ${\upsigma }_{\mathrm{S}}$ and ${\upsigma }_{\mathrm{T}}$ are, respectively, the mean HMI displacement of the surrounding tissues, the mean HMI displacement of the tumor, the standard deviation of the HMI displacement of the surrounding tissues, and the standard deviation of the HMI displacement of the tumor.

## Discussion

We investigated the feasibility of HMI for in vivo breast tumor detection and classification in humans. We were able to characterize tissue types based on the relative HMI displacements quantitatively. The transverse width of the focused beam was under a millimeter (3-dB focal width: 0.24 mm). Therefore, the applied radiation force was highly localized, and tissue properties could be measured with high spatial precision. Using radiation force with a separate transducer, HMI provides sufficient energy to probe stiff and deep-seated breast tumors even if a highly attenuating mass is along the wave propagation path, a limitation inherent to shear wave elastography. In addition, the acoustic radiation force generated by the FUS results in constant harmonic motion within the tissue, which improves the signal to noise ratio (SNR), compared to other elastography techniques. Since HMI can be performed in real-time^50^, parameters such as acoustic intensity and pulse duration can be adjusted or tailored for each tumor in the future to increase the classification accuracy. Real-time HMI with the possibility of delineating tumors against the stromal tissue in addition to conventional B-mode can be used for needle biopsy guidance at the point of care. The tumor infiltration into adjacent tissue and the intraductal spreading assessment are of great importance during breast-conserving surgery^51^. Unlike MRI taken in a prone position, the supine real-time HMI scans can be used to delineate surgery margins during intra-operative applications on supine-positioned patients^45^.

We found that malignant tumors corresponded to higher contrast on the HMI displacement maps compared to benign tumors, which is in accordance with other breast elastography studies^31,52^. In addition, malignant tumors appeared larger on the displacement maps relative to the B-mode images (Figs. 3c, 4c). This might be due to the infiltration of cancer cells at the tumor’s periphery or desmoplastic tissue response, resulting in the resistance of the adjacent tissue to deformation^53–55^. The CNR values (Table 1) were found to be lower for the benign tumors compared to the malignant tumors, except for subjects 8 and 10 who were diagnosed with fibroadenoma. This might be due to the compression of the fibroadenoma to the surrounding tissues, which makes this benign breast mass as the most frequent source of false-positive findings in SWE^56^. Some patients underwent both in vivo and ex vivo (tissue scanned after excision) scans with the same setup. However, localizing the same imaging plane was challenging due to the deformation of the ex vivo breast tissue. It was observed that the contrast between the tumor and the surrounding tissues was higher ex vivo (Figs. 3, 4, 5). Considering the intrinsic nonlinear mechanical behavior of breast tissue due to the higher constraints in vivo, changes in the boundary conditions and lack of physical constraints in the ex vivo specimens could lead to the contrast difference^57^. Future investigations will be performed to identify contributing factors better.

Eight out of ten patients had heterogeneously dense breasts that did not hinder the HMI imaging. Variability across subjects such as age, heterogeneity of breast tissue based on fat and fibroglandular tissue proportions, and menstrual status impacts tissue stiffness^57^ and, consequently, the HMI displacements, which will be studied in the future with a larger statistical population. The ratio of the HMI displacement in the pathological tissue to that of the normal tissue (${\mu }_{T}/{\mu }_{S})$ within the same subject can be estimated and interpreted similar to breast imaging reporting and data system (BI-RADS) classification system. According to Table 1, the ${\mu }_{T}/{\mu }_{S}$ ratio was found to be higher than 0.4 for all the benign tumors (except for subject 8). In order to have more quantitative assessment of the viscoelastic properties and compare the results among other techniques, the Young’s modulus can be estimated based on the HMI displacements by using directional filters to extract the shear waves^58,59^. However, estimation of the Young’s modulus from HMI displacements is out of the scope of this study due to the computationally costly procedures. Previous studies have shown increase in tumor stiffness and consequently decrease in HMI displacement as malignant tumor progresses^44^. In this study, no correlation was found between the displacement and the size of the tumors (Fig. 6), indicating that the displacements were proportional to the stiffness of the tissue and were not affected by the size of the masses. These results are consistent with a simulation study demonstrated that HMI displacements are directly related to the stiffness of any lesion larger than twice of the FUS focal region^47^. Nevertheless, a larger statistical sample size is needed to investigate further the dependence of the displacements on the tumor size. The effect of neoadjuvant therapy on the softening of the tumors was not investigated herein. The change in the HMI displacement can also be used to assess the response of the solid tumors to neoadjuvant therapy, as shown in pre-clinical studies^44,49,59^. According to the histological reviews of all the ex vivo specimens in this study and our previous studies^44,48,49^, no adverse pathological effects, structural nor thermal damage, were observed from exposure to HMI. Additionally, in the clinical setting, no report of discomfort or pain or any adverse event was received from the patients.

Although the clinical prototype of HMI was sufficient to demonstrate the clinical feasibility and the proof-of-concept, we encountered some limitations in this study. The 3-dB beamwidth of the FUS transducer is in the submillimeter order (0.24 mm), which enables a pin-point probing of tissue. The entire ROI was imaged in a raster scan format to allow image reconstruction from each measurement, which improves the lateral resolution of HMI. However, the raster scans were performed with a step size of 2–3 mm as a trade-off to reduce the duration of the scans. The mechanical movement is a relatively slow process, mainly because the robotic arm needed to be paused for a few seconds at each point to become stable. A multi-element FUS transducer will be used in future studies to replace the mechanical movement with the electronic sweeping of the focal spot^60^. In that case, an interval time (of the order of ms) is required between applying each force to avoid shear wave propagation interference between measurements. However, this time duration is deemed negligible compared with the time needed for the mechanical movement of the transducers. It is important to mention that lower displacement regions were obtained on the rightmost half of the displacement maps (e.g., Figs. 3c or 4c). This might be due to the non-slip boundary condition and slight dragging of the breast tissue by mechanical movements of the robotic arm from left to right that can be eliminated by applying electronic steering as well. As the acoustic radiation force decreases away from the focal spot of the transducer, lower displacements were estimated along the depth which were corrected to some degrees (“Acoustic force normalization”). An example is shown in Fig. 2c where deep regions under the tumor appear as stiff tissue due to their low displacements. However, the displacement in those regions is highly affected by the force gradient as a function of the distance from the FUS focus. This characteristic shows the high spatial resolution of the force, but on the other hand, it may limit the imaging field-of-view. These issues can be resolved using a multi-element transducer and electronically steering the beam in the axial direction. In vivo implementation of steered FUS beam in HMI requires further experiments to compensate for the pressure gradient and focal spot shape change as a function of the steering distance, which goes beyond the scope of this study. Four out of 22 patients with relatively shallow or superficial tumors were excluded from the study due to the fixed geometric focus of the FUS transducer (Fig. S2). This a not a fundamental limitation of the HMI technique and imaging at a wide range of depths can be achieved using a different transducer.

A slight breast compression was needed to suppress the fibrocollagenous septa similar to the conventional ultrasound exam to provide an imaging window of the ROI^61^. However, similar to other elasticity imaging techniques, pre-compression must be avoided^62^. We minimized the pre-compression of the tissue during the raster scan, but the tissue compression might be slightly changed, especially during inhaling. The diaphragm moves up to 1.5 cm, and the chest circumference changes 0.7 cm during normal breathing^63^ in the supine position. Since the HMI displacements were at a specific frequency, the respiratory and cardiac motion artifacts were reduced (Fig. S1). In addition, high frame rate acquisition (1,000 frames s^−1^) and short imaging time (80 ms) were faster than the breathing rate. Cross-correlation was used in the axial and lateral directions to correct for in-plane large-scale motion artifacts. However, using the 2-D phased imaging array, we were not able to correct for the out-of-plane motion. Implementation of HMI with a 3-D imaging probe can help to overcome this limitation in future studies. Co-registration of histopathology results with HMI imaging planes would be less challenging using the 3-D imaging probe in order to estimate the HMI measurements within the perilesional tissue and further evaluate the effect of tissue collagen content on the HMI displacements. As discussed later in Methods (“HMI data acquisition”), a phased-array imaging probe with a center frequency of 2.5 MHz was used to acquire the RF data. The transducer could track the induced displacements at a depth of 55 mm without blocking the FUS beam, and its aperture plane could be placed coincident with the central opening of the FUS transducer. However, a pediatric phased-array with similar aperture size and higher center frequency could be utilized for a higher resolution.

In summary, the clinical feasibility of HMI was demonstrated to characterize in vivo breast masses in human subjects. Inducing highly localized oscillations, HMI was capable of differentiating malignant and benign tumors based on the relative displacements. Post-surgical breast specimens from the same patients were imaged with a similar setup to validate the measurements**.** Unlike alternative breast imaging modalities, there is no radiation exposure risk in using HMI and because it is an ultrasound-based technique, it can be repeated in a wide range of patients. The results of this feasibility study indicate the clinical applicability of HMI for human breast imaging and characterization of suspicious masses. This can potentially improve diagnostic specificity and consequently, reduce invasive biopsy rate. Future studies will focus on improving the current clinical system, such as the in vivo implementation of electronic steering, real-time data processing, and development of a clinical harmonic motion imaging-guided focused ultrasound (HMIgFUS) system for treatment planning, monitoring, and assessment.

## Methods

### Experimental protocol

#### In vivo human breast elasticity imaging

**Figure 7:**
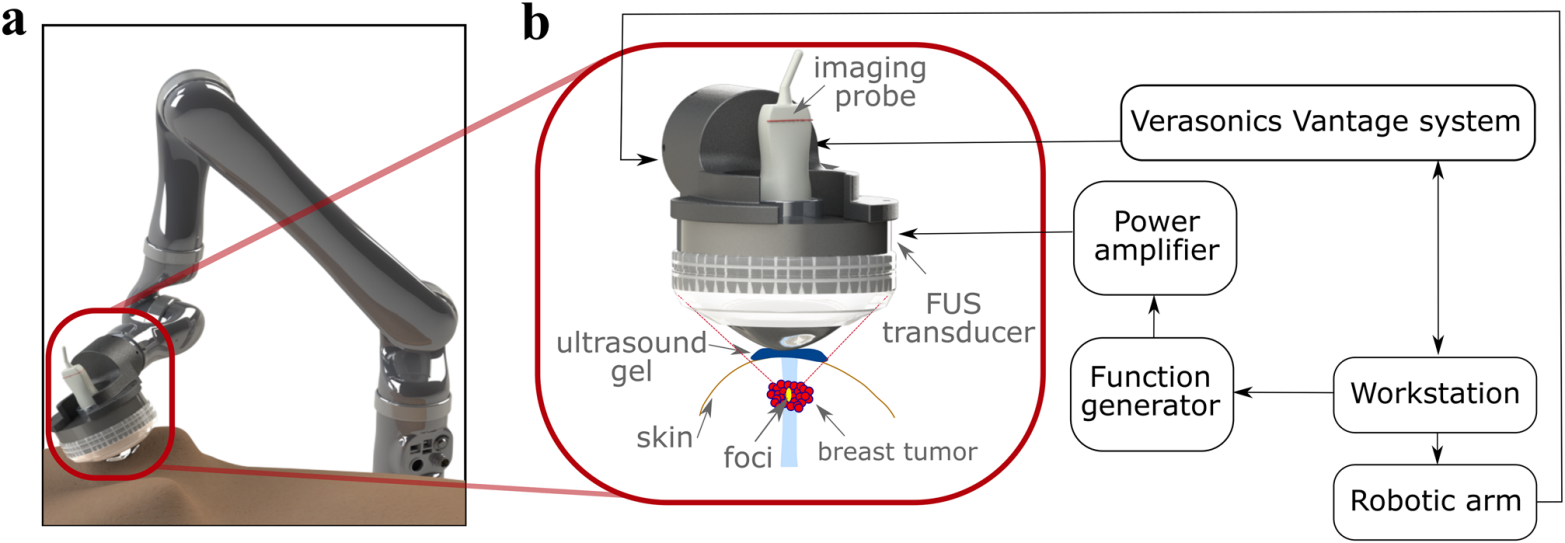
Schematic of the clinical harmonic motion imaging (HMI) setup. (**a**) Positioning of the patient and HMI transducers. (**b**) Block diagram of HMI data acquisition.

The human subject study was conducted under a protocol approved by the institutional review board (IRB) of Columbia University and was carried out in accordance with IRB guidelines and regulations. Informed consent was obtained from all participants. Female patients (age > 18) with the following criteria were approached: (1) palpable breast mass visible on US image and (2) symptomatic breast mass visible on US image. Lactating women, women with breast implants, and women with a history of laser or radiation therapy were not included in the study. Total patient preparation and scan time spanned within 20 min and did not hinder or delay in the standard of care of the patients enrolled. In total, 22 patients enrolled in the study. Seven subjects were excluded as the lesions could not be localized on the B-mode images. One patient was excluded due to the radiotherapy treatment. Four additional subjects were excluded because of the location of the tumors, which were too deep or superficial. The ideal target lesion upper boundary was within 10 mm from the skin due to the fixed position of the radiation force focal spot that will be explained later (the subjects’ population and exclusions are summarized in the Supplementary Materials). Additional subjects (n = 6) were imaged for training purposes, optimization of the parameters including acoustic pressure/intensity, and clinical setup arrangements such as positioning of the subjects and acoustic coupling between the transducer and the subjects (Fig. 7a). HMI images were acquired from all eligible patients before their scheduled US-guided needle biopsy (n = 4) or breast surgery (n = 6). The patient characteristics are summarized in Table 2. Data from 10 patients were used for the study, including patients with adenosis (n = 1), fat necrosis (n = 1), fibroadenoma (n = 2), mature adipose tissue (n = 1), invasive ductal carcinoma (IDC) (n = 3) and invasive lobular carcinoma (ILC) (n = 2). The mean age of the cohort was 60.2 ± 16.4 years. Subjects had a BI-RADS score of 3 (n = 1), 4 (n = 5) and 6 (n = 4).

**Table 1:** CNR ratio of the HMI displacement in the pathological tissue to that of the normal tissue (${\mu }_{T}/{\mu }_{S})$ and mean correlation coefficient values within the tumor (CC_T_) and surrounding tissues (CC_S_).

	Subject 1	Subject 2	Subject 3	Subject 4	Subject 5	Subject 6	Subject 7	Subject 8	Subject 9	Subject 10
CNR	3.53	3.01	6.65	6.65	4.64	7.94	2.14	5.55	3.83	5.31
(${\mu }_{T}/{\mu }_{S})$	0.45	0.66	0.31	0.05	0.24	0.18	0.60	0.26	0.32	0.54
CC _T_	0.9995	0.9979	0.9999	0.9971	0.9982	0.9993	0.9952	0.9999	0.9989	0.9997
CC_S_	0.9982	0.9997	0.8753	0.9981	0.9984	0.9995	0.9995	0.9998	0.9992	0.9999

**Figure 8:**
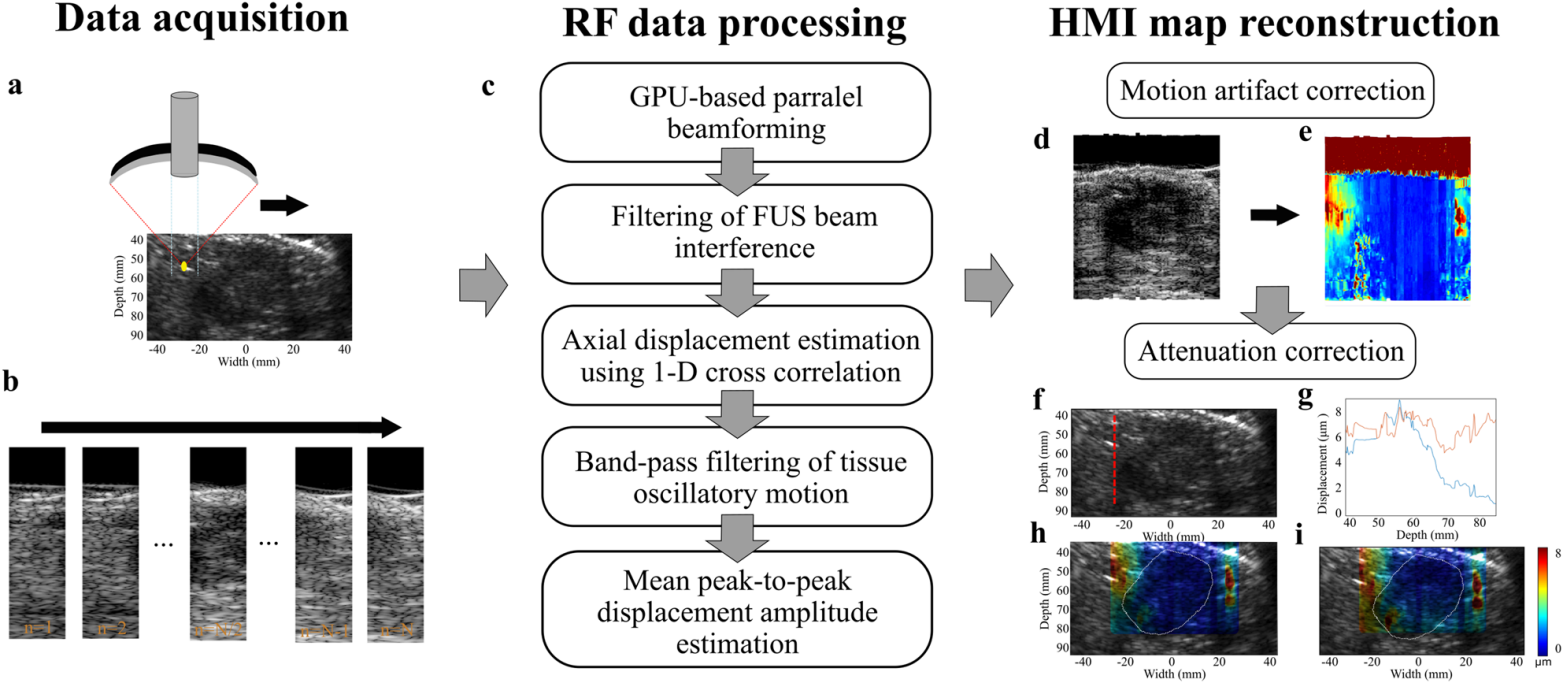
Flowchart of HMI data acquisition, processing, motion correction, and attenuation correction. (**a**) HMI transducers were moved mechanically in a 1-D point-by-point raster scan regimen. (**b**) RF data were acquired at each point. (**c**) RF data were processed offline according to the processing pipeline. (**d**) Large-scale motion artifacts were corrected by applying 2-D cross-correlation on the RF data in the lateral and axial directions (**e**) HMI displacement values were co-registered based on the motion artifact correction. A 2-D displacement map was reconstructed accordingly. (**f**, **g**) Displacement values along the axial direction (red dashed line) were fitted into two exponential curves to correct for acoustic force attenuation above and below the FUS transducer focus. The blue and red lines show the displacement values along the axial direction before and after attenuation correction, respectively. (**h**) Overlaid HMI displacement map on the B-mode image before attenuation correction. (**i**) Overlaid HMI displacement map on the B-mode image after attenuation correction.

The patients were positioned in the supine position (Fig. 7a). A radiologist or trained sonographer located the breast masses using a clinical US scanner (center frequency 6–15 MHz, linear array, Logic E9, GE Healthcare, Milwaukee, WI, USA). A region of interest (ROI) ranging from 40 to 60 mm was chosen based on the size of the tumor. The HMI transducers (described in “HMI data acquisition” section) were placed on top of the breast, where a thin layer of gel was used between the transducer assembly and the tissue to provide acoustic coupling. The transducer assembly was connected and moved mechanically using a robotic arm (JACO2, Kinova inc., Boisbriand, Quebec, Canada) controlled by a PC workstation^64^. The patients were asked to remain immobile with shallow breathing during imaging to reduce the effect of physical movements. Then, a 1-D point-by-point HMI raster scan in the lateral direction was performed in the sagittal plane (dorsal–ventral view) with a step size of 2–3 (Fig. 8a–b). The robotic arm was paused for 3 s at each point prior to data acquisition to settle during mechanical movements. At each point, the induced harmonic motion of the tissue was imaged, and the channel data was stored for offline processing (Fig. 8). The development of real-time processing and steering capability using multi-element FUS transducer is currently ongoing.

**Table 2:** Patient characteristics.

	Age	Tumor size (cm)	Diagnosis	Mammogram impression	Breast density	Neoadjuvant therapy
Subject 1	52	1.4	Adenosis	BI-RADS 4	–	
Subject 2	53	4.5	Fat necrosis	BI-RADS 4	Heterogeneously dense	
Subject 3	72	0.9	Invasive Ductal Carcinoma	BI-RADS 6	Heterogeneously dense	x
Subject 4	89	1.9	Invasive Lobular Carcinoma	BI-RADS 6	–	x
Subject 5	82	2.2	Invasive Ductal Carcinoma	BI-RADS 6	Heterogeneously dense	x
Subject 6	52	4	Invasive Ductal Carcinoma	BI-RADS 4	Heterogeneously dense	
Subject 7	39	2.6	Mature fibroadipose tissue	BI-RADS 4	Heterogeneously dense	
Subject 8	47	3.7	Fibroadenoma	BI-RADS 3	Heterogeneously dense	
Subject 9	67	2.2	Invasive Lobular Carcinoma	BI-RADS 6	Heterogeneously dense	x
Subject 10	49	1.6	Fibroadenoma cellular	BI-RADS 4	Heterogeneously dense

#### Ex vivo human breast elasticity imaging

All mastectomy/lumpectomy specimens were collected from patients with their informed consent who underwent an HMI scan prior to surgery. Tissue collection and handling were approved by the IRB of Columbia University and were carried out in accordance with IRB guidelines and regulations. Immediately after surgical resection, the specimens were transported to the laboratory in phosphate-buffered saline (PBS) for imaging and then returned to the Department of Pathology at Columbia University Medical Center for standard evaluation. The specimens were immersed in a tank of degassed PBS, where a layer of sound-absorbing material was at the bottom to reduce undesired echoes. HMI transducers attached to the robotic arm were moved into the PBS bath, perpendicular to the specimen, and an ROI similar to the one in the pre-surgery HMI image was chosen. An HMI raster scan with the same parameters as the pre-surgery scan was performed on the tissue. A total of 5 specimens from patients with fibroadenoma (n = 1), IDC (n = 3) and ILC (n = 1) were imaged.

### HMI data acquisition

The block diagram of HMI data acquisition is shown in Fig. 7b. A FUS transducer was driven in phase by an amplitude-modulated (AM) signal to generate an oscillatory acoustic radiation force. The single element FUS transducer (center frequency 4 MHz, H-215; Sonic Concepts Inc. Bothell WA, USA) was used to generate the acoustic radiation force at a fixed depth (55 mm from the imaging transducer). A calibration in water using an optical fiber hydrophone (HFO-690, Onda, Sunnyvale, CA, USA) revealed the 3-dB width and length of the focus as 0.24 and 1.19 mm, and the acoustic pressure was measured as 8.47–9 MPa. The AM sinusoidal waveform (AM frequency 25 Hz) was generated by a dual-channel arbitrary waveform generator (AT33522A; Agilent Technologies Inc., Santa Clara, CA, USA) and amplified by a 50-dB gain power amplifier (325LA, Electronics & Innovations (E&I), Rochester, NY, USA). An oscillatory displacement, namely HMI displacement, was induced by the oscillatory radiation force at the focal region of the transducer in the underlying tissue. This oscillatory motion was tracked using an ultrasound imaging transducer confocally aligned through the central opening of the FUS transducer. For this study, a 64-element phased-array imaging probe (center frequency 2.5 MHz, P4-2; ATL Ultrasound, Bothell, WA, USA) was used to track the tissue displacement. FUS exposure was 80-ms long at each point inducing 4-cycle oscillations at 50 Hz, during which 80 frames of RF data were recorded at 1,000 frames s^-1^, using a plane-wave sequence. The FUS transducer was triggered by a Vantage Research scanner system (Verasonics Inc., Kirkland, WA, USA) to synchronize FUS exposure and channel data acquisition.

### Data processing

#### HMI displacement estimation

Processing of the channel data was performed offline in MATLAB (The MathWorks, Natick, MA, USA) as follows: First, a GPU-based delay-and-sum beamforming method was used to reconstruct the beam-formed RF data. Second, the FUS beam interference with the RF signal was removed by a digital notch filter (frequency 4 MHz) followed by a digital low-pass filter (frequency 2.5 MHz). Third, the incremental axial displacement was estimated using a fast, normalized 1-D cross-correlation between every other RF frame with a correlation window length of 0.67 mm and a 95% overlap^65^. Fourth, a band-pass filter (cut-off frequencies 30 and 70 Hz) was applied along the temporal dimension to extract the 50-Hz displacements. Fifth, the peak-to-peak amplitude displacements were averaged over the 4 cycles of oscillations. The processing pipeline is briefly described in Fig. 8c.

#### Motion artifact correction

Large-scale motion was an inevitable artifact in the HMI scans due to the respiratory motion of the patients and mechanical movements of the robotic arm. The transducers were solely moved in the lateral direction with a step size of 2–3 mm. A small step size provided an overlap between the RF signals acquired at two consecutive points. However, due to motion artifacts, a slight change of the step sizes and possible axial movements of the transducer assembly were expected. A 2-D cross-correlation between the beam-formed RF signals at the first frame of a reference point and those of the next point was performed with a lateral window size of 7.67 mm and an axial window size of 30–40 mm (the entire axial field of view). The lateral window size was chosen to be larger than the raster scan step size and yet smaller than the aperture of the P4-2 imaging transducer. The process was repeated by sliding the correlation window laterally with an overlap of 0.47 mm. The distance between the RF signals that yielded the highest correlation coefficient was considered as the corrected lateral step size. The matched RF signal segments were interpolated linearly by a factor of 10 in the lateral direction to increase the precision of the following step. Then, a 2-D cross-correlation with an axial window size of 1.23 mm and a lateral window size equal to the width of the segments was performed and repeated by sliding the correlation window axially with an overlap of 0.19 mm. The criteria for selecting the axial correlation window was upward movements of the diaphragm during respiration, which is estimated to be about 0.5–1.5 cm between points^63^. Any axial movement of the transducers was estimated accordingly. From the mean peak-to-peak displacement map of each point, an ROI centered at the displacement focal zone (“HMI displacement estimation”) with a width of the corrected lateral step was chosen and axially co-aligned to that of the next point based on the estimated axial motions. Therefore, the 2-D displacement maps were reconstructed by co-registering the displacement map segments from all the points (21–31 points) (Fig. 8d–e).

#### Acoustic force normalization

The focus of the FUS transducer was at a distance of 55 mm from the imaging transducer. Lower displacement values were estimated away from the transducer focus as the acoustic force decreased rapidly. In order to compare the displacement values axially, we corrected for this issue and assumed the same force along the axial direction. The focal zone on the peak-to-peak displacement map of one of the raster scan points corresponding to the non-cancerous background tissue was found using a focal spot localization technique^50^. The axial displacement profiles of multiple lateral locations within the focal zone were averaged. Assuming homogeneity of the background breast tissue, two exponential fits were used to correct for the radiation force gradient, above and below the focal spot (Fig. 8f–i). The two fits were applied to all the axial displacement profiles in the final 2-D HMI map to correct for the acoustic radiation force gradient. Similar approaches were applied for displacement image normalization using ARFI imaging^66^. However, there are limitations associated with these approaches based on the assumption of tissue homogeneity, which is not valid in cancerous tissues. Normalization of the force in the lateral direction was deemed unnecessary since the width of the displacement map segment of each point in the final 2-D image was less than the 3-dB displacement focal zone for that point.

### Statistical analysis

Prism 7 (GraphPad Software, La Jolla, CA, USA) was used for the statistical analysis. A two-tailed paired Student’s t-test was used to determine a significant difference between pairwise comparisons. Multiple comparisons were analyzed using a one-way analysis of variance (ANOVA). Pearson Correlation was used to determine the relationship between tumor size and displacements. In all the statistical tests, the null hypothesis was rejected at the 0.05 level.

### Histopathology

The freshly excised breasts were immediately fixed in 10% phosphate-buffered formalin at room temperature for 24 h, followed by a standard process for paraffin embedding. The tissues were then sectioned into 4-µm slides and stained using hematoxylin and eosin (H&E).

## Supplementary information

Supplementary file1
