# Supplementary material for: Harmonic motion imaging of human breast masses: an in vivo clinical feasibility
Source: Sci Rep. 2020 Sep 17;10:15254. doi: 10.1038/s41598-020-71960-5 (PMC7498461; doi:10.1038/s41598-020-71960-5)
Supplement: Supplementary file 1 — Supplementary file1 [file 41598_2020_71960_MOESM1_ESM.pdf]

Supplementary Materials

Harmonic Motion Imaging of human breast masses – an *in vivo* clinical feasibility

Niloufar Saharkhiz[1], Richard Ha[2], Bret Taback[3], Xiaoyue Judy Li[1], Rachel Weber[1], Alireza Nabavizadeh[1], Stephen A. Lee[1], Hanina Hibshoosh[4], Vittorio Gatti[1], Hermes A.S. Kamimura[1], Elisa E. Konofagou[1,2*]

[1]Department of Biomedical Engineering, Columbia University, New York, NY, USA

[2]Department of Radiology, New-York-Presbyterian/Columbia University Medical Center, New York, NY, USA

[3]Department of Surgery, New-York-Presbyterian/Columbia University Medical Center, New York, NY, USA

[4]Department of Pathology and Cell Biology, New-York-Presbyterian/Columbia University Medical Center, New York, NY, USA

* Correspondence may be addressed to EEK (ek2191@columbia.edu).

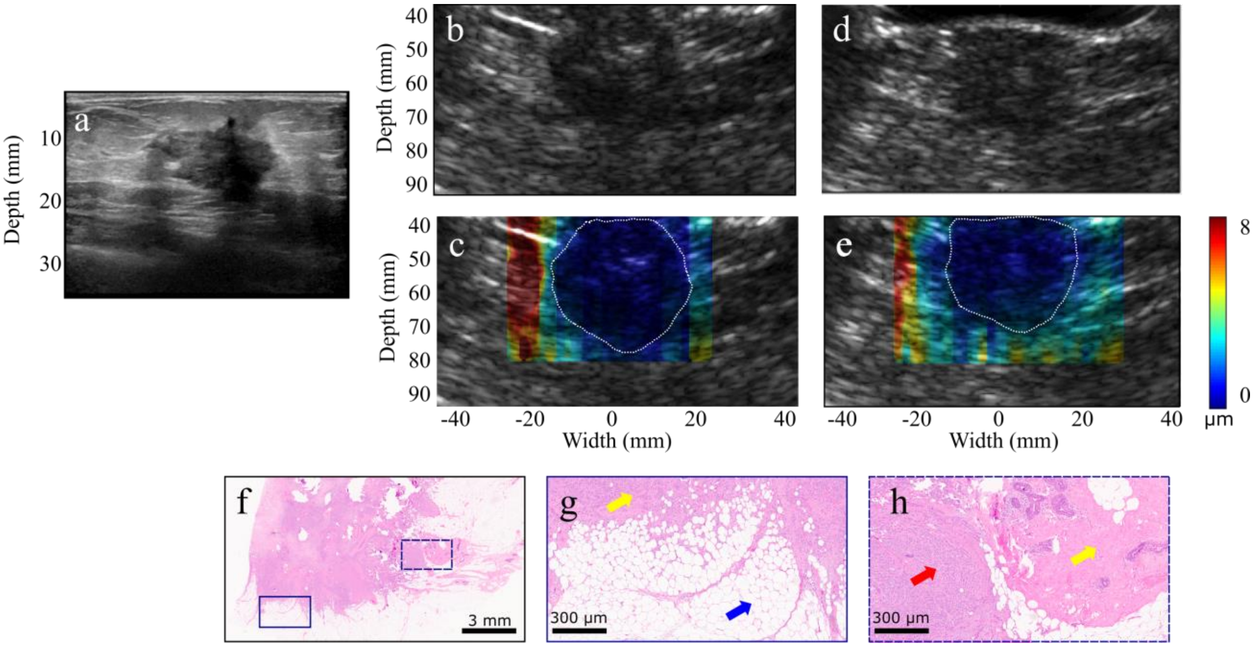

Fig. S1. An 89-year-old subject diagnosed with invasive lobular carcinoma (ILC) was imaged at a similar imaging plane before and after general anesthesia using the same acquisition setup and parameters to validate the reproducibility of the HMI estimations and the effect of breathing. (a) Breast clinical US image of the patient. (b) B-mode image of the tumor captured using the 2.5-MHz imaging transducer before resection. (c) HMI displacement map overlaid on the B-mode. (d-e) B-mode image and overlaid HMI displacement map acquired after general anesthesia of the same patient (f-h) Hematoxylin and eosin stained section of the tumor. The Red arrows show invasive carcinoma, the yellow arrow shows fibrous normal breast tissue and the blue arrow shows mature adipose tissue.

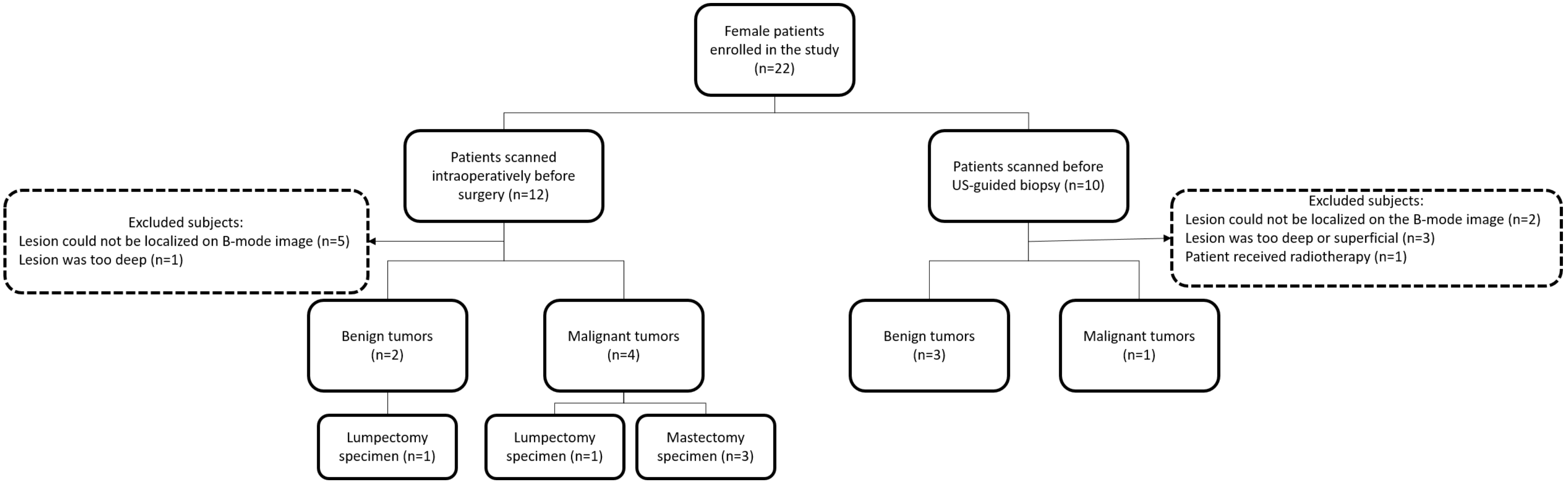
Female patients enrolled in the study (n=22)
Patients scanned intraoperatively before surgery (n=12)
Patients scanned before US-guided biopsy (n=10)
Excluded subjects:
Lesion could not be localized on B-mode image (n=5)
Lesion was too deep (n=1)
Excluded subjects:
Lesion could not be localized on the B-mode image (n=2)
Lesion was too deep or superficial (n=3)
Patient received radiotherapy (n=1)
Benign tumors (n=2)
Malignant tumors (n=4)
Benign tumors (n=3)
Malignant tumors (n=1)
Lumpectomy specimen (n=1)
Lumpectomy specimen (n=1)
Mastectomy specimen (n=3)

Fig. S2. Flowchart of all the participants in the study and exclusions.
